# Gas Classification Using Deep Convolutional Neural Networks

**DOI:** 10.3390/s18010157

**Published:** 2018-01-08

**Authors:** Pai Peng, Xiaojin Zhao, Xiaofang Pan, Wenbin Ye

**Affiliations:** 1 School of Electronic Science and Technology, Shenzhen University, Shenzhen 518060, China; pengpai_sh@szu.edu.cn (P.P.); eexjzhao@szu.edu.cn (X.Z.); 2 School of Information Engineering, Shenzhen University, Shenzhen 518060, China; eexpan@szu.edu.cn; 3 Key Laboratory of Optoelectronic Devices and Systems of Ministry of Education and Guangdong Province, College of Optoelectronic Engineering, Shenzhen University, Shenzhen 518060, China

**Keywords:** gas classification, deep convolutional neural networks, electronic nose

## Abstract

In this work, we propose a novel Deep Convolutional Neural Network (DCNN) tailored for gas classification. Inspired by the great success of DCNN in the field of computer vision, we designed a DCNN with up to 38 layers. In general, the proposed gas neural network, named GasNet, consists of: six convolutional blocks, each block consist of six layers; a pooling layer; and a fully-connected layer. Together, these various layers make up a powerful deep model for gas classification. Experimental results show that the proposed DCNN method is an effective technique for classifying electronic nose data. We also demonstrate that the DCNN method can provide higher classification accuracy than comparable Support Vector Machine (SVM) methods and Multiple Layer Perceptron (MLP).

## 1. Introduction

The electronic nose, which imitates the perceptual mechanisms found in biological olfactory organs, has been widely used in many applications for a variety of purposes including medicine and diagnostics [1], food production [2] and environment monitoring [3,4]. A conventional electronic nose typically consists of three parts: a gas sensor array that is a set of chemical sensors which convert the chemical smell signal to an electronic signal; a preprocessing block that extracts the features of the gas signal; and a pattern recognition system that can recognize the olfactory features of the substance being tested. In this paper, we focus upon just the third part.

In the past several decades, many pattern recognition algorithms have been proposed for gas classification. With regard to electronic nose applications, the *K*-Nearest Neighbor (KNN) method was introduced in [5,6]. This method is quite simple and effective, but involves the storing of training data. As a result, this is not a suitable approach when memory is limited. In order to overcome this shortcoming, a Gaussian Mixture Model (GMM) approach was proposed for odor classification in [7]. In this case, transient features are used that offer the possibility of real-time classification. In [8,9,10], a multiple layer perception (MLP) network is used to address the nonlinearity present in odor classification. However, in this case, the need for a full connection can cause the classifier to get trapped in overfitting, leading to a bad prediction. A Support Vector Machine (SVM), by contrast, is an advanced supervised classification technique that outperforms Artificial Neural Network (ANN) models, particularly when only a small data-set is available for training [11,12]. In [13], a recently developed machine learning technique, the Relevance Vector machine (RVM), is proposed instead. An RVM is similar to an SVM but requires fewer kernel functions. In [14], deep Restricted Boltzmann Machines (RBM) are proposed as a way of using electronic noses to identify bacteria in blood. Similarly, Liu et al. [15] suggest that gas recognition under drift might be accomplished by using a deep Boltzmann Machine and a Sparse Auto-Encoder (SAE). However, these two models are rather, when compared to the Deep Convolutional Neural Networks (DCNNs), used in computer vision. With RBM and SAE, the depth is usually 3–4 layers, with 1–2 of them being hidden, as opposed to the dozens to hundreds layers offered by DCNNs. Moreover, RBM and SAE are unsupervised learning techniques. In other words, they try to learn a hidden representation pattern, with the learned features then being fed into another classification framework, making feature extraction/learning and classification quite distinct.

In this paper, an end-to-end (DCNN) called GasNet that is tailored for gas classification is proposed. The motivation of adopting DCNN is that the DCNN has the ability to jointly learn the features and classification boundaries directly from raw input data. In the DCNN architecture, the convolutional kernels work as filters that can automatically extract the features of the raw input data. The performance of classical approaches such as SVM and MLP is very related to the prior knowledge and human effort in feature extraction. However, for a certain gas dataset, to find a good feature extraction method is already a difficult task and needs much prior knowledge of that dataset. The proposed GasNet consists of: six convolutional blocks; a pooling layer; and a fully-connected layer. Each convolutional block that consists of six layers, i.e., two convolutional layers, two batch normalization layers and two Rectified linear unit (ReLU) layers are proposed to extract highly representative features and thus the proposed DCNN is up 38 layers. Together, these various layers make up a powerful deep model for gas classification. Compared with previously proposed methods, this DCNN has two particularly significant advantages. Firstly, because SVM, KNN, and GMM are all typical conventional machine learning algorithms, a DCNN is better at mining and extracting effective features. This is thanks to the large amount of nonlinear activation neurons in the network architecture. Secondly, a DCNN is an end-to-end deep network, while RBM and SAE break the whole framework into two independent parts, i.e., feature learning and gas classification. An end-to-end approach offers better performance because there is less information loss. The contribution of this paper can be summarized as follows:

(1) We propose a very deep convolutional neural network called GasNet for gas classification. To the best of our knowledge, our work is the first one to exploit a real “deep” learning-based model to recognize gas types.
(2) The proposed CNN contains up to 38 layers. This offers huge advantages over conventional machine learning algorithms (e.g., SVM, KNN, and GMM) and other recently proposed more shallow networks (e.g., RBM and SAE).
(3) Experimental results confirm that GasNet achieves higher classification accuracy than other previous approaches.

The rest of this article is organized as follows. In the next section, the proposed GasNet is presented. Section 3 discusses the experimental results that compare the performance of the classification accuracy of different classifier methods. Some concluding remarks are given in Section 4.

## 2. The Proposed GasNet Tailored for the Gas Classification

In this section, we describe the proposed GasNet DCNN in more detail. First of all, we provide an overview of GasNet and how it is suitable for gas classification. We then elaborate upon each component/layer in the network architecture. After this, we present the network training schedule.

### 2.1. GasNet Overview

Deep learning is the process of training a (deep) neural network given a specific task. A neural network is nothing but a large and complex mathematical function with millions of parameters. Thus, deep learning has great power to learn the decision function by feeding in input and expected output (we call training data). Creating or “training” this function functionality requires a large amount of training data because of the huge number of parameters. At the beginning of the training process, the parameters are set to random values (or values generated with a normal distribution). Then, for each data example in the training set, the loss (or error) value is computed as the difference between the expected output (what we call ground truth) and predictions. After that, the values of the parameters are slightly changed so as to decrease the loss by using an effective schedule called a Stochastic Gradient Descent (SGD). This process is repeated for every data example in the training set many times over. As a result, the neural network is able to learn decision functions, assuming enough training data is provided.

In recent years, we have witnessed a revolutionary trend in the field of computer vision. DCNNs have become the state-of-the-art for visual recognition and understanding. The key to the success of DCNNs is the convolutional operation or convolutional layer, which can automatically learn useful implicit features according to the intensity of pixels in input images. The key to the success of DCNN is the convolutional operation or convolutional layer that is able to automatically learn useful implicit features from the intensities of the pixels in the input images. The ImageNet challenge [16] has been especially notable in how it has contributed to the birth of a variety of effective DCNN architectures such as AlexNet [17] (ImageNet 2012 winner), Zeiler-Fergus (ZF)-Net [18] (ImageNet 2013 winner), Visual Geometry Group(VGG)-Net [19] (ImageNet 2014 winner), ResNet [20] (ImageNet 2015 winner) and Inception series [21,22]. However, they cannot be applied to gas classification directly because, first of all, existing DCNNs are specifically designed for processing visual data. As a result, the input is usually a high-dimensional image with three channels (in the RGB color space), e.g., 299×299×3. The dimensionality of features collected by gas sensors, however, is quite a bit lower, e.g., each sensor generates a 16D feature vector. Another issue is that the cost of obtaining large scale visual data is much lower than it is for gas data. Finally, the pre-trained weights (parameters) of DCNNs for visual data are publicly available while there are no pre-trained weights available for gas data (Our work is the first one to exploit DCNNs to recognize gas types). Because of these issues, in order to leverage deep learning for recognizing gas types, we have had to carefully design a novel DCNN that can accept gas feature vectors as input and then output a gas class label. The nuts and bolts of the deep network architecture are covered in the next section.

### 2.2. Network Architecture

Overall, GasNet is composed of convolutional, batch normalization pooling operations. For the sake of feature representational power, a few convolutional and batch normalization operations together make up a computational unit that we call “convolutional block”. In the rest of this section, we describe the overall network architecture and then detail the convolutional block components.

#### 2.2.1. Overall Architecture

**Figure 1:**
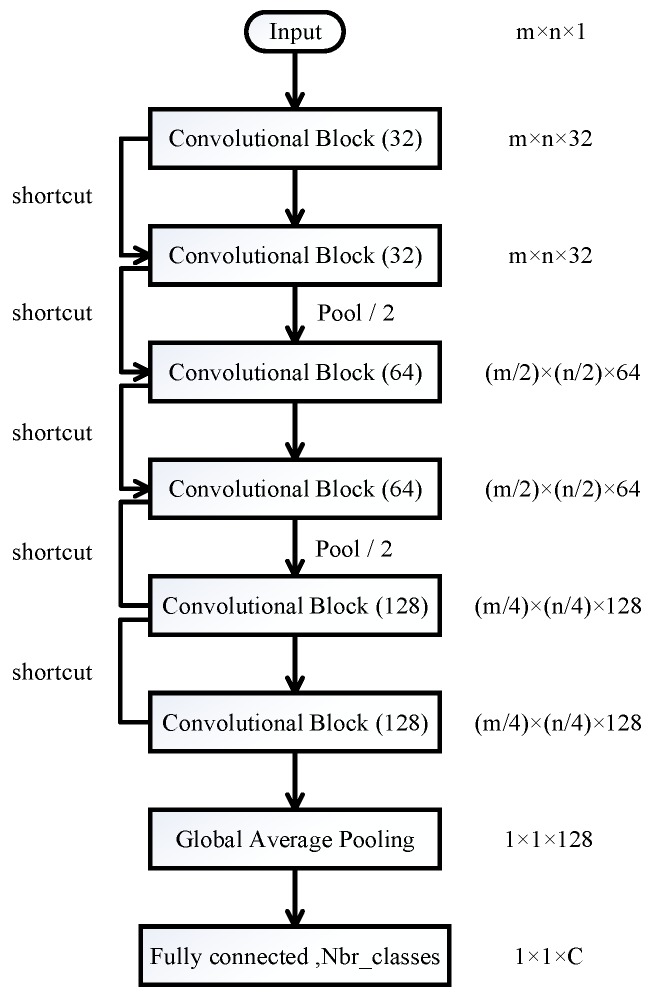
GasNet architecture.

Figure 1 depicts the proposed overall network architecture. It begins with an input layer, which accepts a feature tensor with the shape of m×n, where *m* is the number of sensors and *n* is the dimensionality for each sensor. Then, a stack of *convolutional blocks* are applied in order to extract useful and highly representative features. Motivated by the design philosophy of VGG-Net [19] and ResNet [20], the network follows two design rules: (1) the number of feature maps keeps the same when the layer’s output shape is preserved; (2) the number of feature maps doubles when the layer’s output shape is reduced by a half. These two design concepts ensure that the computational cost is roughly the same for each convolutional block. Overall, GasNet includes three max-pooling layers and each reduces the output resolution by a half. A *global average pooling* layer is followed by the last convolutional block. A global average pooling operation is usually used to distill the crucial information while removing redundancy. Finally, GasNet ends with a fully connected layer with number of types of neurons and Softmax activation. Note that, inspired by [20], we add shortcut (or skipped connection) for convolutional blocks. This helps to avoid any “gradient vanishing” problem. In other words, the gradients will flow back through the shortcut during back-propagation, so that the gradients can skip over the adjacent convolutional block to reach the next one.

In summary, the input tensor shape for GasNet is m×n×1, followed by six convolutional blocks separated by two max-pooling operations. The output tensor shape becomes $\frac{m}{4}\times \frac{n}{4}\times 128$ right after the last convolutional block. Next, the global average pooling layer averages the activations for each feature map and outputs a tensor with shape of 1×1×128. Lastly, the GasNet outputs *C* values for each input vector and each represents the probability of being that specific gas type.

#### 2.2.2. Convolutional Block

**Figure 2:**
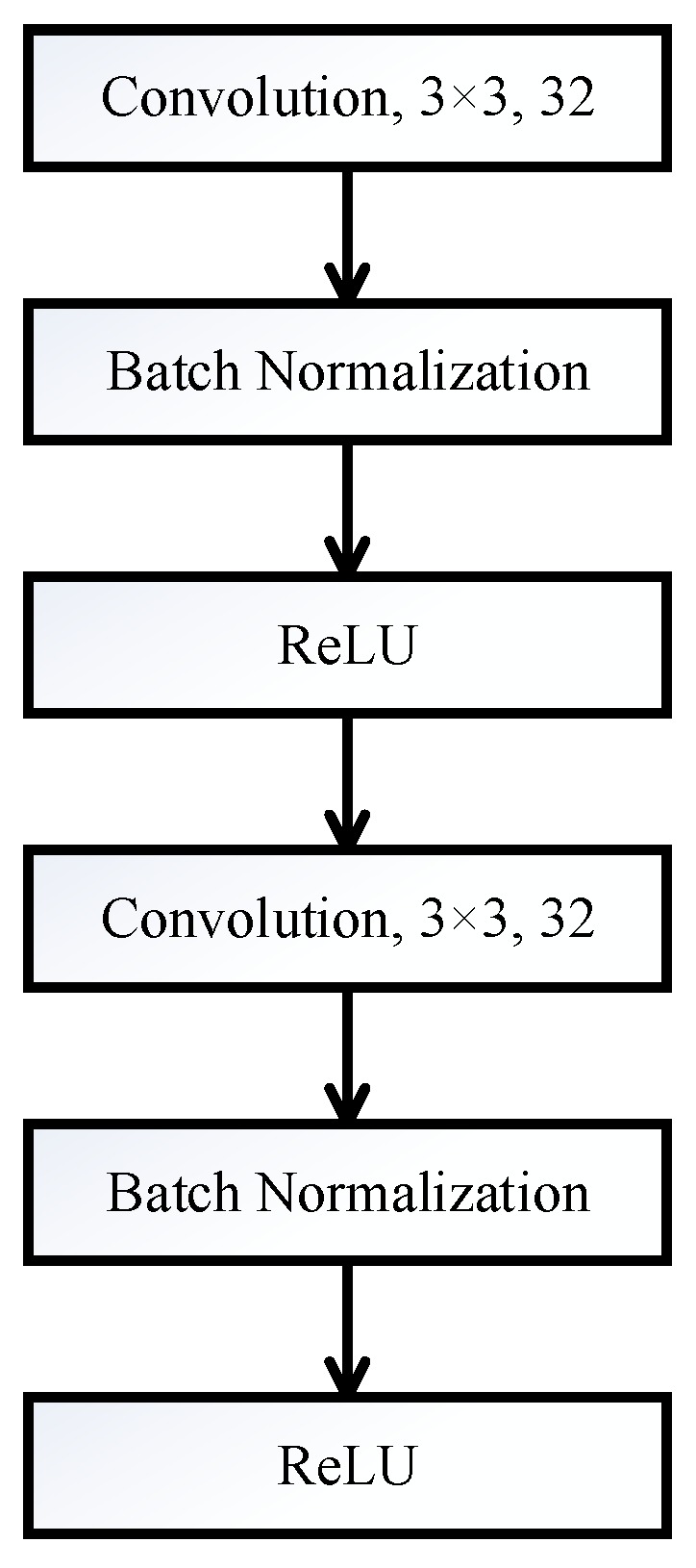
Convolutional block.

**Figure 3:**
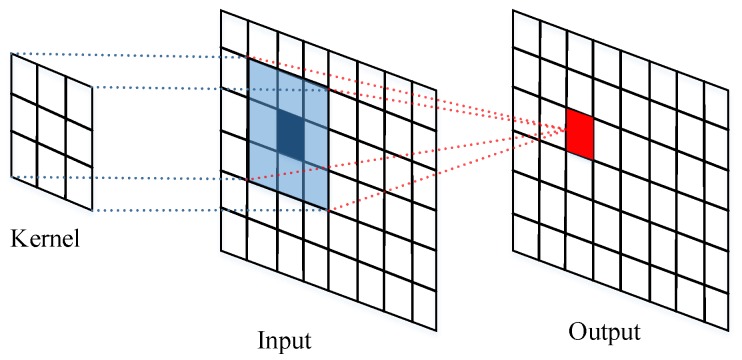
Convolution operation illustration.

As shown in Figure 2, each convolutional block consists of two typical convolutional layers and each one is followed by a *batch normalization* (BN) [23] layer and ReLU activation function. The convolutional operation is illustrated as Figure 3. Convolution is the process of adding each element of the 2D matrix to its local neighbors, weighted by the kernel as shown in Figure 3. Assume that input matrix *A* has dimensions (Ma,Na) and kernel matrix *B* has dimensions (M,N). When the block calculates the full output size, the equation for the 2D discrete convolution can be expressed as

(1) $$C\left(i,j\right)=\sum_{m=0}^{Ma-1}\sum_{n=0}^{Na-1}A\left(m,n\right)\times B\left(i-m,j-n\right),$$

where 0≤i<Ma+M−1 and 0≤j<Na+N−1. BN is a useful recently proposed technique that can potentially help in two ways, by providing: faster learning and higher overall accuracy. Frequently, normalization (i.e., shifting inputs to zero mean and unit variance) is used as a pre-processing step to make data comparable across features. As the data flows through a deep network, the weights and parameters adjust these values, sometimes making the data too big or too small again. This is a problem that is referred to as “internal covariate shift”. BN is used to normalize the data in a mini-batch during the network training process. The convolutional kernel size is fixed to 3×3 and padding is 1 so that the output shape is the same as the input within one block.

In our view, the convolutional block we have designed has two advantages: (1) it introduces more nonlinearity into the network by stacking two consecutive convolutional layers; (2) the number of parameters is limited due to having 3×3 kernels, so stacking more convolutional blocks to increase the depth of the network is feasible. Suppose the input kernel size is 3×3 and the number of input channels and output channels are both *c*. Then, a single output feature map contains 3×3×c parameters. Note that we have *c* feature maps. Thus, the total number of parameters is 3×3×c×c, which is $9c^{2}$. Thus, one single convolutional block contains $18c^{2}$ (parameters for BN could be omitted).

### 2.3. Network Training

**Loss Function** Cross-entropy is frequently used for loss/error calculations in classification tasks in deep learning. Ideally, the error should be 0 if the deep network (the classifier) is perfectly classifying an unseen data sample during the testing stage. Failing this, some error (between 0 and 1) is computed as a punishment on the classifier. As the network encounters more and more data, it tends to predict more and more precisely. If we let *y* be the ground truth label for a certain data point *x* and $\hat{y}$ is the prediction generated by the classifier. The cross-entropy error for this specific data point is defined as follows:

(2) L(y,y)^=−∑i=1Cyilogy^i,

where *C* is the number of classes in the dataset. It is worth noting that the overall loss is the averaged loss over all the training data.

**Optimization** For our DCNN, we use Stochastic Gradient Descent (SGD) as the optimization method. The initial learning rate is set at 0.01 and the momentum at 0.9. We decrease the learning rate by a factor of 0.1 if the accuracy stops increasing for five epochs until it reaches 0.00001. In order to prevent from overfitting, we also use an early stop strategy. All network weights were initialized following the suggestions proposed in [24], i.e., variance of neurons in the network should be 2/n (*n* is the number of neurons in the layer).

## 3. Experimental Results

**Figure 4:**
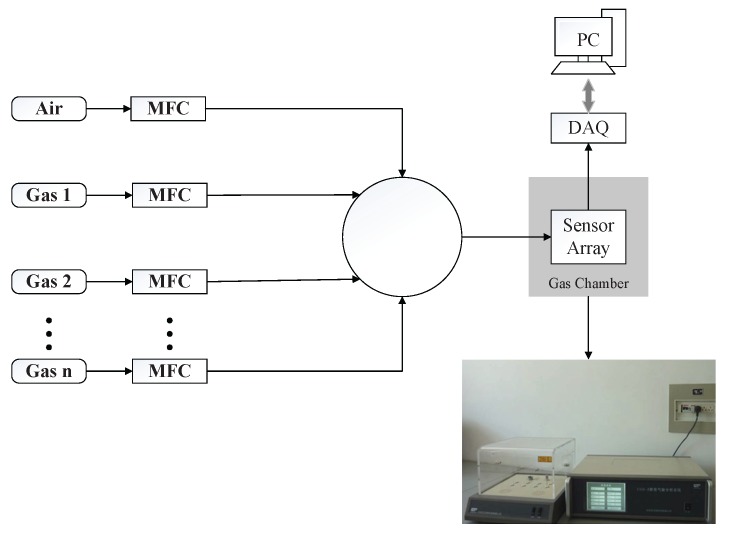
Experimental setup to acquire signatures of the target gases with the sensor array.

The automated gas delivery setup used to acquire response data of target gases with the sensor array is shown in Figure 4. Eight commercial Figaro metal oxide semiconductor (MOS) sensors (Arlington Heights , IL, USA) are used to build the gas sensor array and their corresponding part numbers are listed in Table 1. The electronic signals of these sensors are simultaneously acquired through a chemical gas sensor CGS-8 system in 10 Hz sampling rate. The flow rate of the target gas can be controlled by the computer controlled mass flow controllers (MFCs) . Through changing the ratio of the flow rate between target gas and background gas, we can get a range of concentrations of target gas. In our experiment, there are 20 concentrations for each gas. For each type of gas at each concentration, we make 15 repeated measurements and thus there are 300 measurements for each type of gas. As there are four types of gases (Carbon Monoxide, Methane, Hydrogen and Ethylene) in our case, we totally have 300×4=1200 samples in our data sets. In our experiment, the measurement for each gas at certain concentration last 100 s. Therefore, for each measurement, there are 100×10=1000 points for each sensor. The gas sensor array consists of eight sensors and thus there are there are 100×10×8=8000 points in each raw sample data. Without any additional feature engineering, the raw sampled data is directed taken as the input of the SVM, MLP and the proposed DCNN method. We randomly split the dataset into 70% training and 30% test sets. In other words, for each type of gas, 300×0.7=210 samples (each sample is 8000×1 column vector) are selected for training the classifiers, while the rest samples are used to test the classifiers. Therefore, the overall training samples is 210×4=840 Since the training set and test set are randomly selected from the whole dataset, to eliminate the bias of the test result, we then repeated this train-test procedure 100 times with different random splits. Then, we average the accuracy of each test to get the accuracy for each classifier.

### 3.1. Baseline Approach

**SVM**—Before the widespread adoption of neural networks in recent years, support vector machines (SVM) [12] were the dominant pattern recognition and classification tool. A great deal of research effort was devoted to exploiting SVMs for gas classification. For this reason, SVM was chosen as one of our baseline approaches. It should be noted that an L-SVM is proposed in [25] . The L-SVM is an ensemble method and can achieve higher classification accuracy than single SVM if there is some drift between the training data and test data, since there is no drift in our dataset and thus the L-SVM method is not compared in our paper. Since the input for an SVM is usually a one-dimensional feature vector, we stretched the input tensor for GasNet by rows. In other words, we reshaped a three-dimensional tensor m×n×1 to a one-dimensional vector m∗n. We trained SVM with an Radial basis function (RBF) kernel. The kernel bandwidth parameter γ and punish function *C* parameter were chosen using a 5-fold cross validation by performing a grid search in the range $\left[2^{-7},2^{-6},\ldots ,2^{6},2^{7}\right]$ and $\left[2^{-7},2^{-6},\ldots ,2^{8},2^{9}\right]$, respectively. In the proposed 5-fold cross-validation, the original sample is randomly partitioned into five equal sized subsamples. A single subsample is retained as the validation data for testing the model, and the remaining four subsamples are used as training data. The cross-validation process is then repeated five times. Then, the five results from the folds are averaged to produce a single estimation.

**MLP**—New research [10] has proposed a real-time gas classification service system that uses a Multi-Layer Perceptron (MLP) artificial neural network to detect and classify gas sensor data. MLP is actually a simple neural network with a hidden layer (excluding the input layer and output layer). In the MLP, the number of hidden layers is 1 and the neurons in each hidden layer is 1024.

### 3.2. Learning Curves

**Figure 5:**
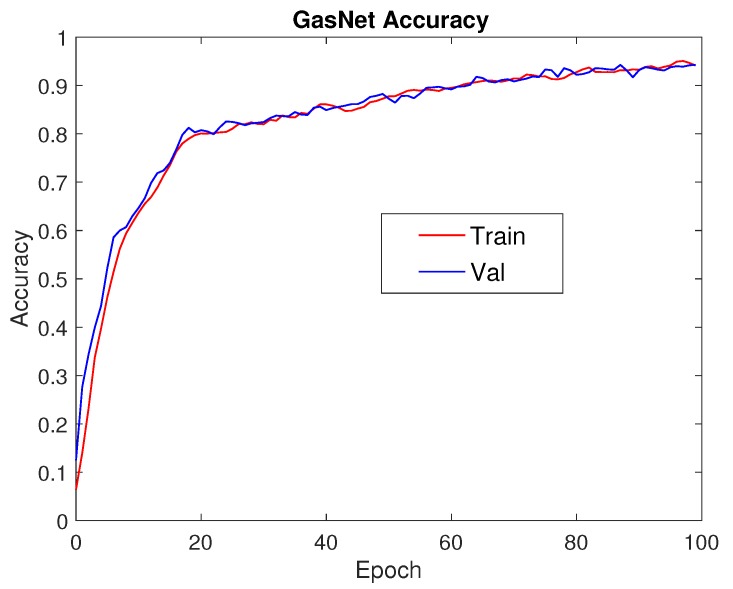
Accuracy.

**Figure 6:**
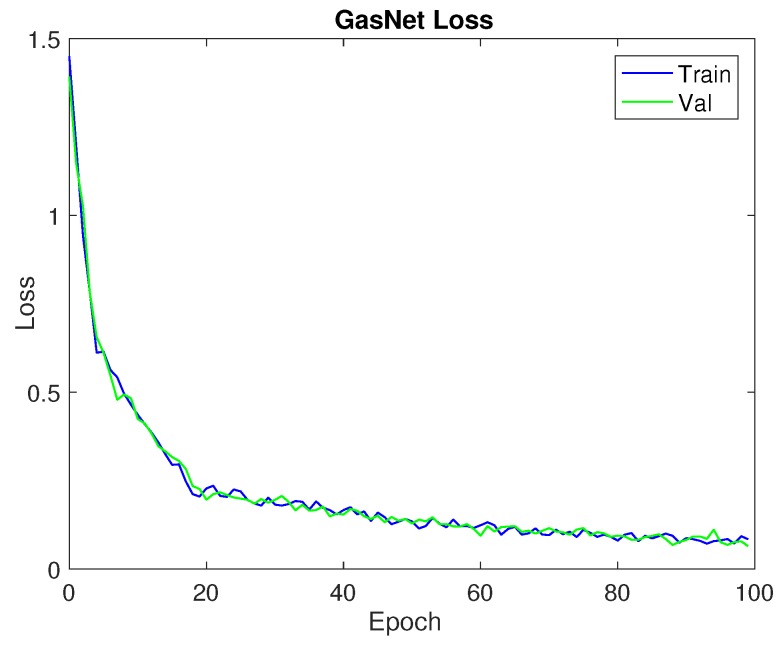
Loss.

Learning curves are one of the most important evaluation measures for machine/deep learning algorithms. As can be seen in Figure 5, training accuracy and validation accuracy maintain a steady increase as the number of training iterations increases. Similarly, in Figure 6, it can be seen that training loss and validation loss continue to decrease as the number of epochs goes up. Based on the learning curves, we can conclude that we didn’t incur an “overfitting” or “underfitting” phenomenon.

### 3.3. Performance of Recognition

Classification/recognition accuracy is one of the most important evaluation metrics for supervised learning algorithms. For this, the number of correctly recognized examples is divided by the total number of testing examples. Recognition accuracy performance is summarized in Table 2. SVM is a typical example of the “shallow learning” school. Thus, it is no wonder that SVM achieves similar performance results to MLP. It can be seen that our proposed GasNet outperforms SVM and MLP by some margin. There are three reasons for this: (1) GasNet is a much deeper neural network compared with SVM or MLP; (2) the convolutional block contains more than one convolutional layer, making GasNet more representative; and (3) the kernel size is tailored for a gas classification scenario, helping the network to learn more expressive and discriminative features.

### 3.4. The Computational Complexity of the DCNN

The computational cost of the machine learning methods can be divided into two parts that is training and estimation. The training time for the different methods is listed in Table 1. And it can be seen that the proposed CNN has the highest computational time in the training part. However, the training computational complexity is not very important, as the training operation is performed offline and in most cases is performed just once. Therefore, in our cases, only the operational computational cost of MLP, SVM and the proposed CNN is compared. The operational computational cost of SVM is O($n_{s}vd$), where $n_{s}v$ is the number of support vectors, *d* is the input data dimension that can be expressed as m×n, *m* is the number of gas sensors and *n* is the dimensionality for each sensor. For the MLP, the computational complexity can be expressed as O($n_{l}kd$), where $n_{l}$ is the number of hidden layers, and *k* is the number of neurons in each layer. As for the proposed CNN, the convolutional operation dominates the overall computational complexity and thus, for the computational complexity estimation, only convolutional operation are taken into account. There are two convolution layers in the proposed convolution block. For each convolution layer, the computational cost is 2 × m × n × M × N × L, where, M × N is the size of the convolutional kernel and L is the number of kernels. Since there are six convolution blocks, the overall computational cost of the proposed CNN can be estimated as O(12d×M×N×L). In our case, the kernel size is 3 × 3, and the kernel number *L* is 32. Generally, the operational computational cost of the proposed CNN is much higher than SVM and MLP. In other words, DCNN achieves the higher classification accuracy at the cost of higher computational complexity than MLP and SVM.

## 4. Conclusions

In this work, we have presented a method for leveraging deep learning for the recognition of gas types. To do this, we designed a novel DCNN that can accept gas feature vectors as input and then output a gas class label. As far as we know, this is the first DCNN that has been used for gas classification. DCNNs are better at mining and extracting effective features because of the large number of nonlinear activation neurons in the network architecture, resulting in higher classification accuracy than that of conventional methods such as SVM or MLP. Our experimental results have served to verify this claim.

## Figures and Tables

**Table 1:** Types of metal oxide semiconductor (MOS) sensors (provided by Figaro Inc. (Arlington Heights, IL, USA)).

Channel	Sensor Part Number
0	TGS821
1	TGS812
2	TGS2610
3	TGS2612
4	TGS3870
5	TGS2611
6	TGS816
7	TGS2602

**Table 2:** Performance of recognition accuracy.

Model	Validation Accuracy	Training Time(s)
SVM	79.9%	2
MLP	82.3%	17
**GasNet**	**95.2%**	154
